# Selection of Pof^-^ *Saccharomyces eubayanus* Variants for the Construction of *S. cerevisiae* × *S. eubayanus* Hybrids With Reduced 4-Vinyl Guaiacol Formation

**DOI:** 10.3389/fmicb.2018.01640

**Published:** 2018-07-27

**Authors:** Jasper A. Diderich, Susan M. Weening, Marcel van den Broek, Jack T. Pronk, Jean-Marc G. Daran

**Affiliations:** Department of Biotechnology, Delft University of Technology, Delft, Netherlands

**Keywords:** 4-vinyl guaiacol, hybridization, genetic, high-throughput screening (HTS), brewing fermentation, ferulic acid, wort sugar

## Abstract

*Saccharomyces pastorianus* is an interspecies hybrid between *S. cerevisiae* and *S. eubayanus*. The identification of the parental species of *S. pastorianus* enabled the *de novo* reconstruction of hybrids that could potentially combine a wide array of phenotypic traits. Lager yeasts are characterized by their inability to decarboxylate ferulic acid present in wort, a phenotype also known as Pof***^-^*** (phenolic off-flavor). However, all known *S. eubayanus* strains characterized so far produce clove-like aroma specific of 4-vinyl guaiacol, a decarboxylated form of ferulic acid. This study explored a non-GMO approach to construct Pof***^-^*** *S. eubayanus* variants derived from the parental strain *S. eubayanus* CBS 12357. To rapidly screen a population of UV-mutagenized cells two complementary assays were developed. The first assay was based on the difference of light absorption spectra of ferulic acid and 4-vinyl guaiacol, while the second was based on the difference of sensitivity of Pof***^-^*** and Pof^+^ strains to cinnamic acid. The *S. eubayanus* variant HTSE042 was selected and was confirmed not to produce 4-vinyl guaiacol. Whole genome sequencing revealed that this variant lost the subtelomeric region of the CHRXIII right arm that carried the two clustered genes *SePAD1- SeFDC1* whose deletion in a naïve *S. eubayanus* strain (CBS 12357/FM1318) resulted in an identical phenotype. Subsequently, the Pof***^-^*** variant was crossed with a Pof^-^ *S. cerevisiae* partner. The resulting hybrid was not able to convert ferulic acid demonstrating the undisputable value of the mutagenized variant HTSE042 to eventually construct *S. cerevisiae* × *S. eubayanus* hybrids with phenotypic characteristics of *S. pastorianus*.

## Introduction

Since its discovery in Patagonia seven years ago (Libkind et al., 2011), *Saccharomyces eubayanus* received significant attention which to a large extent was related to its role in the reconstruction of the interspecies hybrid lager brewing yeast, *S. pastorianus* (Nakao et al., 2009; van den Broek et al., 2015; Okuno et al., 2016). Particularly, the *S. eubayanus* CBS 12357 was used in crosses aiming at the formation of artificial *S. cerevisiae* × *S. eubayanus* hybrids (Hebly et al., 2015; Krogerus et al., 2015, 2016; Mertens et al., 2015; Magalhaes et al., 2017) exhibiting novel phenotypic characteristics. Although *S. eubayanus* CBS 12357 is not able to consume all wort sugars, in particular maltotriose (Hebly et al., 2015; Krogerus et al., 2015), it exhibits several brewing traits suitable for lager brewing including growth at a temperature as low as 4°C (Hebly et al., 2015), proficient maltose utilization and formation of desired aroma molecules (Mertens et al., 2015). These characteristics might then be transferred to hybrids holding significant value for the brewing industry (Krogerus et al., 2017a). However, such hybrids recurrently showed formation of a clove-like, also referred to as medicinal flavor during wort fermentation (Krogerus et al., 2016, 2017b). While a clove-like aroma is highly desirable in German “hefeweizen-style” beers (Gallone et al., 2016), this characteristic is considered an off-flavor (phenolic off-flavor (Pof)) in lager beer.

The main source of the clove-like off-flavor in lager beer is the aromatic compound 4-vinyl guaiacol (4-VG). Malted barley grains that serve to prepare brewing wort are a source of a wide range of phenolic acids (*e.g.*, p-hydroxybenzoic acid, vanillic acid, chlorogenic acid, *p*-coumaric acid, ferulic acid) (Yu et al., 2001) which possess potential health benefits (Dykes and Rooney, 2007). Among them, ferulic acid, the precursor of 4-VG is associated with arabinoxylan essentially found in endosperm aleurone cells of cereal grains (Wilson et al., 2006). During malting and more specifically during the germination step feruloyl esterases are expressed and release feruloyl moieties from the arabinoxylan enabling subsequent hydrolysis of the polysaccharide chain by specialized enzymes (Vanbeneden et al., 2007). While a fraction of the phenolic off-flavors in beer originates directly from the wort, or through chemical conversion as a consequence of temperature and oxygen (*e.g.*, during wort boiling or aging in the bottle) (McMurrough et al., 1996), a significant fraction results from the enzymatic conversion of ferulic acid into 4-VG by the fermenting yeast (Coghe et al., 2004). Initially only *PAD1* (phenylacrylic acid decarboxylase) was associated with 4-VG formation (Clausen et al., 1994), a gene previously identified as *POF1* that was implicated in phenolic off-flavour (Pof) in beer (Meaden and Taylor, 1991). A second ferulic acid decarboxylase gene (*FDC1*) was later characterized (Mukai et al., 2010). Both *PAD1* and *FDC1* form a functional gene cluster at the end of chromosome IV in *Saccharomyces cerevisiae*. The functional characterization of these genes revealed that both encoded enzymes are essential to express a ferulic acid decarboxylase activity (Mukai et al., 2010). However, contrasting with the initial premise, *PAD1* would encode a flavin mononucleotide (FMN)-bearing protein which does not function as a decarboxylase. Rather, *PAD1* would contribute to the formation of a novel cofactor necessary for the *FDC1* encoded decarboxylase activity (Lin et al., 2015) similarly to UbiX function in *E. coli* (White et al., 2015). The phenolic acid decarboxylase encoded by *FDC1* was shown to have a broad substrate range that in addition of ferulic acid also included cinnamic acid (McKenna et al., 2014), coumaric acid (Bhuiya et al., 2015) and caffeic acid (Huang et al., 2012) which suggests that *PAD1-FDC1* plays a more general role in detoxification of phenolic acids (Baranowski et al., 1980; Clausen et al., 1994; Larsson et al., 2001; Richard et al., 2015). However in spite of this important function, sequence analysis of a large cohort of *S. cerevisiae* strains revealed that the Pof phenotype was highly strain dependent and has been strongly influenced by domestication. Mostly unwanted in alcoholic beverages, the absence of 4-VG production in sake, wine, and most beer types fermenting strains was accompanied by occurrence of loss-of-function mutations in *PAD1* and *FDC1*. In contrast wild type, baker’s and bioethanol strains most systematically contained functional alleles (Gallone et al., 2016). Similarly, the wild type *S. eubayanus* CBS 12357 harbors functional copies of the two genes (Libkind et al., 2011; Baker et al., 2015; Hebly et al., 2015). The typical lager brewing *S. pastorianus* hybrids (Nakao et al., 2009; van den Broek et al., 2015) have been selected for their Pof***^-^*** phenotype which was acquired during the intensive domestication history of these yeasts. As a result *S. pastorianus* Frohberg strains [*e.g.*, WS34/70 (Nakao et al., 2009), CBS 1483 (van den Broek et al., 2015)] have lost the *S. eubayanus* genes caused by a translocation between the right arms of *S. cerevisiae* CHRIV and *S. eubayanus* CHRXIII. Simultaneously *S. cerevisiae FDC1* orthologue carries a frameshift in the first half of the gene (position 521/1501) resulting in the introduction of a premature stop codon, while the *S. cerevisiae PAD1* gene although full length harbors two non-synonymous mutations resulting in replacement of His38 and Ala47 by Tyr and Val respectively (van den Broek et al., 2015). Therefore the reconstruction of artificial hybrids representative of modern industrial *S. pastorianus* brewing strains would require Pof***^-^*** parental strains. While multiple Pof^-^ *S. cerevisiae* strains have been characterized (Gallone et al., 2016), hitherto no Pof^-^ *S. eubayanus* strains have been described. Although yeast strain design has been enhanced by the emergence of genome-editing tools such as CRISPR-Cas9 (Jinek et al., 2012; Doudna and Charpentier, 2014), techniques that enable highly accurate genetic alterations such as gene deletion, *in vivo* site directed mutagenesis and novel genes chromosomal integration (DiCarlo et al., 2013; Mans et al., 2015; Jakociunas et al., 2016; de Vries et al., 2017) are available, but, consumer concerns about use of genetically modified (GM) organisms for food and beverage production preclude the use of these approaches in industry (Varzakas et al., 2007).

The objective of the present study was to construct *S. eubayanus* strains that lost their capacity to produce 4-VG in order to form Pof^-^ *S. pastorianus* artificial hybrids. To select 4-VG negative *S. eubayanus* variants from an UV-mutagenized cell suspension, a novel multi-read-out screening assay combining an assessment of growth in presence and absence of cinnamic acid and a spectrophotometric assay screening the conversion of ferulic acid was developed. Positively screened single cell lines were phenotypically and genotypically characterized and used in crosses with Pof***^-^*** *S. cerevisiae* strains.

## Materials and Methods

### Yeast Strains

The strains used in this study are listed in **Table 1**. Working stock cultures were cultivated in YPD medium (10 g⋅L^-1^ Bacto yeast extract, 20 g⋅L^-1^ Bacto peptone and 20 g⋅L^-1^ glucose) until mid-exponential phase, completed with sterile glycerol [final concentration 20% (v/v)] and stored at -80°C as 1 mL aliquots until next inoculation.

**Table 1:** Strains used in this study.

Strain name	Yeast species	Genotype	Reference
CBS 12357/FM1318	*S. eubayanus*	*MATa/MATα SePAD1-SeFDC1/SePAD1-SeFDC1)*	Libkind et al., 2011
IMK747	*S. eubayanus*	*MATa/MATα Sepad1-Sefdc1Δ::amdS/SePAD1-SeFDC1*	This study
IMK749	*S. eubayanus*	*MATa/MATα Sepad1-Sefdc1Δ::amdS/Sepad1-Sefdc1Δ::amdS*	This study
HTSE022	*S. eubayanus*	*MATa/MATα SePAD1-SeFDC1/SePAD1-SeFDC1* (UV mutagenised)	This study
HTSE023	*S. eubayanus*	*MATa/MATα SePAD1-SeFDC1/SePAD1-SeFDC1* (UV mutagenised)	This study
HTSE033	*S. eubayanus*	*MATa/MATα SePAD1-SeFDC1/SePAD1-SeFDC1* (UV mutagenised)	This study
HTSE037	*S. eubayanus*	*MATa/MATα SePAD1-SeFDC1/SePAD1-SeFDC1* (UV mutagenised)	This study
HTSE042	*S. eubayanus*	*MATa/MATα Sepad1-Sefdc1Δ/ Sepad1-Sefdc1Δ^∗^* (UV mutagenised)	This study
IMK439	*S. cerevisiae*	*MATα SUC2 MAL2-8^c^ ura3Δ::KanMX*	Gonzalez-Ramos et al., 2013
CEN.PK113-7D	*S. cerevisiae*	*MATa SUC2 MAL2-8c*	Salazar et al., 2017
CEN.PK122	*S. cerevisiae*	*MATa/MATαSUC2/SUC2 MAL2-8c/ MAL2-8c*	Entian and Kötter, 2007
CBS 1483	*S. pastorianus*	Prototrophic wild-type lager brewing yeast	(van den Broek et al., 2015)
FRY153	*S. cerevisiae*	*3n*	Hebly et al., 2015
IMS0408	*S. eubayanus* × *S. cerevisiae*	*MATa/MATα Scura3Δ::KanMX/SeURA3 Scpad1-ScFDC1/ SePAD1-SeFDC1Δ*	Hebly et al., 2015
HTSH009 (HTSE042 × IMK439)	*S. eubayanus* × *S. cerevisiae*	*MATa/MATα Scura3Δ::KanMX/SeURA3 Scpad1-ScFDC1/ Sepad1-Sefdc1Δ^∗^*	This study
HTSH010 (HTSE042 × IMK439)	*S. eubayanus × S. cerevisiae*	*MATa/MATα Scura3Δ::KanMX/SeURA3 Scpad1-ScFDC1/ Sepad1-Sefdc1Δ^∗^*	This study
HTSH011 (HTSE042 × IMK439)	*S. eubayanus* × *S. cerevisiae*	*MATa/MATα Scura3Δ::KanMX/SeURA3 Scpad1-ScFDC1/ Sepad1-Sefdc1Δ^∗^*	This study
HTSH012 (IMK749 × IMK439)	*S. eubayanus* × *S. cerevisiae*	*MATa/MATα Scura3Δ::KanMX/SeURA3 Scpad1-ScFDC1/ Sepad1-Sefdc1Δ::amdS*	This study
HTSH013 (IMK749 × IMK439)	*S. eubayanus* × *S. cerevisiae*	*MATa/MATα Scura3Δ::KanMX/SeURA3 Scpad1-ScFDC1/ Sepad1-Sefdc1Δ::amdS*	This study
HTSH014 (IMK749 × IMK439)	*S. eubayanus* × *S. cerevisiae*	*MATa/MATα Scura3Δ::KanMX/SeURA3 Scpad1-ScFDC1/ Sepad1-Sefdc1Δ::amdS*	This study


*^∗^Indicates *pad1-fdc1* deletion resulting from UV mutagenesis.*

### Media and Growth Conditions

Batch cultivations in shake flasks, 24-well and 96-well microtiter plates were performed in synthetic wort medium (SWM) that contained 14.4 g⋅L^-1^ glucose, 2.3 g⋅L^-1^ fructose, 85.9 g⋅L^-1^ maltose, 26.8 g⋅L^-1^ maltotriose, 5 g⋅L^-1^ (NH_4_)_2_SO_4_, 3 g⋅L^-1^ KH_2_PO_4_, 0.5 g⋅L^-1^ MgSO_4_⋅7H_2_O, 1 mL⋅L^-1^ trace element solution, 1 mL⋅L^-1^ vitamin solution and supplemented with the anaerobic growth factors ergosterol and Tween 80 (0.01 g⋅L^-1^ and 0.42 g⋅L^-1^ respectively), as previously described in (Verduyn et al., 1992). For screening of UV mutagenized colonies SWM was supplemented with either cinnamic acid (SWM-ca) or ferulic acid (SWM-fa) at a final concentration of 1 mM. The 0.5 M stock solutions of ferulic acid and cinnamic acid were prepared in 100 % ethanol. When the amdSYM cassette was applied as selection marker, strains were plated on synthetic medium with acetamide (SM-Ace) which contained 3 g⋅L^-1^ KH_2_PO_4_, 0.5 g⋅L^-1^ MgSO_4_⋅7H_2_O, 0.6 g⋅L^-1^ acetamide, 6.6 g⋅L^-1^ K_2_SO_4_, 1 mL⋅L^-1^ of a trace element solution and 1 mL⋅L^-1^ of a vitamin solution (Solis-Escalante et al., 2013).

Interspecific hybrids issued from the crosses of *S. eubayanus* spores and *S. cerevisiae* MATa IMK439 (Gonzalez-Ramos et al., 2013) were selected on SMUG containing 3 g⋅L^-1^ KH_2_PO_4_, 0.5 g⋅L^-1^ MgSO_4_⋅7H_2_O, 0.5 g⋅L^-1^ urea and 1 g⋅L^-1^ glutamate, 1 mL⋅L^-1^ of a trace element solution and 1 mL⋅L^-1^ of a vitamin solution (Verduyn et al., 1992) and supplemented with 200 μg⋅mL^-1^ of G418 antibiotic (Wach et al., 1994).

The pH in all the media was adjusted to 6.0 with KOH. Solid media were prepared by adding 2 % (w/v) agar to the media described above.

Diploid yeast strains were sporulated in sporulation liquid medium (SLM) containing 2.5 g⋅L^-1^ yeast extract, 15 g⋅L^-1^ KCH_3_CO_2_, 20 g⋅L^-1^ potassium acetate and 2.5 g⋅L^-1^ glucose. The pH of SLM was adjusted to 7.0 with KOH.

### Construction of Double *FDC1* and *PAD1 S. eubayanus* Deletion Strains

To construct a *S. eubayanus* 4-VG negative strain, the clustered genes Se*PAD1-SeFDC1* were deleted. For this, a deletion cassette including the recyclable amdSYM-cassette was constructed. The amdSYM was amplified from the plasmid pUG-amdSYM (Solis-Escalante et al., 2013) using the primers 9461 and 9462 which harbored homology to the *FDC1* promoter and to the *PAD1* terminator respectively (**Table 2**). PCR amplification was performed using Phusion Hot Start II High Fidelity Polymerase (Thermo Scientific, Waltham, MA, United States) according to the manufacturer instructions using HPLC purified, custom synthesized oligonucleotide primers (Sigma Aldrich, Zwijndrecht, Netherlands) in a Biometra TGradient Thermocycler (Biometra, Gottingen, Germany). The deletion cassette was subsequently isolated from a 1% (w/v) agarose gel using Zymoclean Gel DNA recovery Kit (Zymo Research Corporation, Irvine, CA, United States).

**Table 2:** Primers used in the study.

Primer	Sequence (5′ to 3′)
9461	CTTGTAGCCATACGTCTTCCAATTTTCGTTAACTTTATCAACTAATTCCTTCGAATACCCCAGCTGAAGCTTCGTACGC
9462	TTCTATTACCTAGAAGAGCTAGTTCAGCTTTATTGAAAACCCCAGGACTCTCTGCAAGATGCATAGGCCACTAGTGGATCTG
9	CGCACGTCAAGACTGTCAAG
9322	GCGGCTGAACATATCTCCTG
9328	CGGCGAAATGCATGGATACG
9350	CAATATTCGACACACCTATGCTG
9351	TAGAATTGTTGACACATGGAATTCC
Scer F2	GCGCTTTACATTCAGATCCCGAG
Scer R2	TAAGTTGGTTGTCAGCAAGATTG
Seub F3	GTCCCTGTACCAATTTAATATTGCGC
Seub R2	TTTCACATCTCTTAGTCTTTTCCAGACG


Exponentially growing *S. eubayanus* CBS 12357 (Libkind et al., 2011) was transformed with the amplified amdSYM-cassette using the LiAc transformation protocol (Gietz and Schiestl, 2007). Cells were plated on SM-Ace plates (Solis-Escalante et al., 2013). The deletion of one of the two copies was confirmed by colony DNA isolation and PCR (Looke et al., 2011) using Dream*Taq* PCR Master Mix (2×) (Thermo Fisher Scientific) with primers, 9322, 9328 and 9 (**Table 2**). After confirmation of the deletion a transformant was re-streaked three times on a SM-Ace plate, and a single colony isolate was stocked as IMK747.

The construction of a homozygote diploid *S. eubayanus* carrying the double *pad1-fdc1*Δ*::*amdSYM mutation was performed through sporulation and tetrad dissection of IMK747. The biomass of an end-exponential culture of the strain IMK747 was collected by centrifugation (5 min., 3000 × *g*) and washed twice with demineralized water. Subsequently the washed biomass was incubated in 20 mL SLM for 72 h at 20°C in an orbital incubator (Infors Multitron, Bottmingen, Switzerland) at 200 rpm. The presence of asci was checked by microscopic observation. The ascus walls were digested with zymolyase (Zymo research; 5 U⋅mL^-1^ Zymolyase in 1 M sorbitol) for 20 min. at 20°C. Tetrads were dissected using a MSM400 dissection microscope (Singer Instruments, Watchet, United Kingdom) and grown on SMG. After replication on SM-Ace, spores exhibiting growth were confirmed to have no *PAD1-FDC1* wild type alleles left by colony PCR as described previously. After three times re-streaking, a single colony isolate was stocked and renamed IMK749. IMK749 was confirmed to be a diploid strain by its capacity to sporulate as described above.

### Generation of *S. eubayanus* Variants by Exposure to UV Light

The diploid *S. eubayanus* strain CBS 12357 was grown in YPD until early stationary phase. Cells were harvested by centrifugation (1000 × *g* at 4°C for 5 min.) and washed with demineralised water. Then, cells were incubated for 72 h at 20°C in SLM. Presence of asci spores was checked by light microscopy. Cell density was determined with a flow cytometer (BD Accuri C6, BD Biosciences, Sparks, MD, United States). Spores were diluted in demineralised water to a final concentration of 200,000 spores mL^-1^. Spores were plated on SWM-agar plates (approximately 20,000 spores per plate) and subjected to UV irradiation (UVC-lamp, 36W, MSC-Advantage Biological Safety Cabinet, Thermo Fisher Scientific) for 80 s yielding a survival rate of circa 1% (Hashimoto et al., 2005) and incubated for 5 days at room temperature in the dark. A set of ca. 2000 mutagenized colonies were colony-picked using a Tecan Freedom Evo 200 (Tecan, Männedorf, Switzerland) equipped with a Pickolo colony picker (Sci Robotics, Kfar Saba, Israel) and arrayed in 96-well microtiter plates filled with 200 μL SWM.

### Screening of Strains for Reduced Ability to Produce 4-VG

Arrayed UV mutagenized isolates were grown in 96-well microtiter plates filled with SWM at 20 °C for 48 h in an orbital incubator (Infors Multitron) at 250 rpm. Subsequently, the microtiter plates were replica-plated in three different media by transferring 10 μl of each culture into fresh microtiter plates filled with either 200 μL SWM or SWM-fa or SWM-ca. The reference strain *S. eubayanus* CBS 12357 was added to column 6 of each microtiter plate as a positive control. Column 5 of each 96-well microtiter plate only contained SWM as control for contamination in between wells. The 96-well microtiter plates were enclosed by sandwich covers and a clamp system (Duetz et al., 2000). The arrayed UV-mutagenized strains were grown for 3 days at 20°C in an orbital incubator (Infors Multitron) at 250 rpm. Growth was determined by measuring the optical density at 660 nm with a Tecan Infinite M200Pro spectrophotometer. The Cinnamic Acid Sensitivity Index (CASI) was calculated as the ratio of the OD_660nm_ of the culture grown in presence of cinnamic acid over the culture without cinnamic acid, after 72 h. Maximum inhibition was set at 100, as follows:

$$CASI=100-\left(\frac{{OD}_{660\,\;nm}culture\,\;with\,\;1\,\;mM\,\;cinnamic\,\;acid}{{OD}_{660\,\;nm}culture\,\;without\,\,\;cinnamic\,\;acid}\times 100\right)$$

Additionally, SWM-fa culture supernatants were collected by centrifugation for 5 min at 2500 × *g* at 4°C. After transfer into a fresh plate, five-fold diluted supernatant absorption spectra between 250 and 400 nm were recorded using a Tecan Freedom Evo 200. For strain comparison the Ferulic Acid Conversion Index (FACI) was calculated as the ratio of the absorbance at 310 nm of the culture grown in the presence of 1 mM ferulic acid after 72 h, over the absorbance of the culture medium with 1 mM ferulic acid at 310 nm. Complete absence of the peak at 310 nm (high ferulic acid conversion) was set at 100, as follows:

$$FACI=100-\left(\frac{{Abs}_{310\,\;nm}culture\,\;with\,\;1\,\;mM\,\;ferulic\,\;acid}{{Abs}_{310\,\;nm}culture\,\;medium\,\,\;with\,\;1\,\;mMferulic\,\;acid}\times 100\right)$$

### Screening of Strains by Principal Component Analysis

Principal component analysis was performed using RStudio, version 1.1.383^1^ (RStudio, Inc., Boston, MA, United States) and R software, version 3.4.3^2^. The softwares Ggbiplot, version 0.55^3^ and ggplot version 2.2.1 (Wickham, 2009) were used to visualize the principal component space.

Principal Component Analysis (PCA) was performed on a dataset comprising growth on SWM and growth in the presence of cinnamic acid after 72 h (as determined by OD_660nm_) and the FACI values at 280, 290, 300, 310, 320, 330, 340, 350, and 360 nm of culture supernatants grown in SWM-fa.

### Characterization of Strains With Reduced Ability to Produce 4-VG

Selected strains were cultivated under micro-aerobic conditions in Duetz-MTPs (Enzyscreen, The Netherlands), consisting of 24-square polypropylene deep-well plates, low-evaporation sandwich covers and extra high cover clamps (Duetz et al., 2000) at 20°C at 250 rpm in an orbital incubator (Infors Multitron) in 3 mL SWM, SWM-ca and SWM-fa.

### Generation of *S. cerevisiae* × *S. eubayanus* Hybrids

Mass mating of *S. cerevisiae* IMK439 (Gonzalez-Ramos et al., 2013) and *S. eubayanus* IMK749 spores was done as previously described in (Hebly et al., 2015). In short, 100 μL of the *S. cerevisiae* MATa IMK439 taken at mid-exponential phase was mixed with *S. eubayanus* IMK749 spores and incubated for 4 h at 30°C in an orbital incubator (Infors Multitron) at 200 rpm before plating on SMUG selective plates. Single colony isolates were allowed to stabilize on SWM for approximately 50 generations before phenotyping and stocking. Three single potential hybrids were selected and restreaked three times on SMUG selective plates. These interspecific hybrid strains were stored and named HTSH012, HTSH013 and HTSH014. Similarly haploid vegetative *S. cerevisiae* IMK439 and spores of *S. eubayanus* HTSE042 (an UV-mutagenized variant of *S. eubayanus* CBS 12357 exhibiting reduced 4-VG production) were mass-mated and plated on SMUG. The resulting hybrids were named HTSH009, HTSH010 and HTSH011. Successful hybridisation was confirmed by PCR using a primer pair specific for *S. cerevisiae* (Scer F2/Scer R2, **Table 2**) and for *S. eubayanus* (Seub F3/Seub R2) as described in Pengelly and Wheals (2013). Further confirmation of the hybridization was performed by flow cytometry and whole-genome sequencing.

### Analytical Methods

Ferulic acid, 4-vinyl guaiacol (4-VG) and cinnamic acid were measured using an Agilent 1260 LC equipped with an 1260 Infinity Diode Array Detector measuring at 214 nm, and an Agilent Zorbax SB-C18 Column (4.6 × 5.0, 3.5 micron) operated at 30°C (Koopman et al., 2012; Vos et al., 2015). A gradient of acetonitrile and 20 mM KH_2_PO_4_ (pH 2) with 1% (v/v) acetonitrile was used as eluent at a flow rate of 0.8 mL⋅min^-1^, increasing from 0–10% acetonitrile in 6 min. followed by an increase to 40% acetonitrile until 23 min. From 23 to 27 min, 100 % 20 mM KH_2_PO_4_ (pH 2) with 1 % acetonitrile was used as eluent. Ferulic acid, 4-VG and cinnamic acid standards for calibration were obtained from Sigma Aldrich. Stock solutions were prepared in 100 % ethanol. Dilutions were made in milliQ water.

Staining of cells with SYTOX Green Nucleic Acid Stain was performed as described by (Haase and Reed, 2002). Stained cells were analyzed on a flow cytometer equipped with a 488 nm laser (BD Accuri C6, BD Biosciences). The hybrids were compared with strains of known ploidy (n/2n, *S. cerevisiae* CEN.PK113-7D; 2n/4n, *S. cerevisiae* CEN.PK122; 3n/6n, *S. cerevisiae* FRY153) (van den Broek et al., 2015).

### Sequence Analysis of Strains

Genomic DNA was prepared as described previously in (Kuijpers et al., 2016). Libraries with an average insert size of 413 bp and 323 bp for *S. eubayanus* CBS 12357 and *S. eubayanus* HTSE042, respectively, were constructed and paired-end sequenced with a read length of 150-bp as described previously (van den Broek et al., 2015).

A total of 21,345,630 and 20,998,964 reads were generated for the strains *S. eubayanus* CBS 12357 and HTSE042, respectively, accounting for more than 3 Gb of data per strain representing a minimum of 125-fold coverage of the diploid genome of *S. eubayanus*. Sequence reads of each strain were mapped onto *S. eubayanus* CBS 12357 [genome PRJNA243390, (Baker et al., 2015)] using the Burrows–Wheeler Alignment tool (BWA) and further processed using SAMtools (Li et al., 2009; Li and Durbin, 2010).

Single-nucleotide variations and indels were determined using Pilon (Walker et al., 2014) based on the BWA.bam output file. The Pilon results file.vcf was visualized using the Integrative Genomics Viewer IGV^4^. Sequence data are available at NCBI^5^ under Bioproject PRJNA472853.

## Results

### Deletion of *SeFDC1* and *SePAD1* Is Sufficient to Abolish 4-VG Production in *S. eubayanus* CBS 12357

**FIGURE 1:**
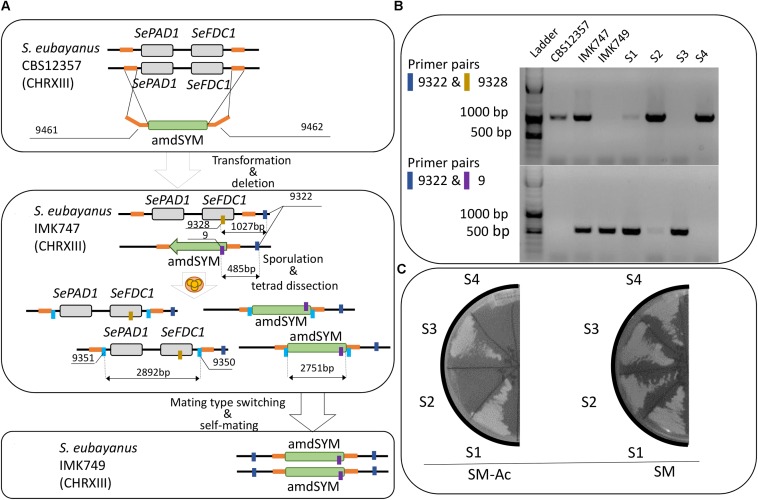
Construction of targeted *PAD1-FDC1* deletion in *Saccharomyces eubayanus* CBS 12357. **(A-1)** The deletion cassette was amplified using the primers pair 3144/3145 and the plasmid pUG-amdSYM (Solis-Escalante et al., 2013) as template. The fragment was then transformed in *S. eubayanus* CBS 12357. **(2)** Transformants were genotyped with primers pairs 9322/9328 and 9322/9 to discriminate between wild type (1027 bp) and deleted (485 bp) loci respectively. Heterozygote diploid IMK747 (*SePAD1-SeFDC1*/*Sepad1-Sefdc1*Δ::amdSYM) was sporulated. After ascus digestion the spores were dissected resulting in a two:two segregation of the *PAD1-FDC1* locus. Spores were germinated. Single colony isolates of acetamide growing spores were selected and checked for ploidy. **(3)** A 2n isolated homozygote (*Sepad1-Sefdc1*Δ::amdSYM/*Sepad1-Sefdc1*Δ::amdSYM) was selected and renamed IMK749. **(B)** PCR analysis was performed to confirm correct integration of the gene disruption cassettes using amdSYM (Solis-Escalante et al., 2013) of the *PAD1-FDC1* locus in *S. eubayanus*. PCR was carried out on reference strain CBS 12357, IMK747, IMX749 and four segregants (S1–S4) of a single tetrad of IMK747. PCRs were performed with the primers pairs 9322/9328 and 9322/9 for *PAD1-FDC1* and *pad1-fdc1*Δ loci, respectively. The amplification of the wild-type *PAD1-FDC1* and *pad1-fdc1*Δ parental strain, of the loci generated fragments of 1027 and 485 bp, respectively. **(C)** The four segregants (S1–S4) of a single tetrad of IMK747 were grown on SM-Ac and SM. The plates were incubated at 30°C and were read after 3 days.

To assess the full dynamic range of 4-VG production in *S. eubayanus* CBS 12357, a homozygote diploid carrying a double *SePAD1-SeFDC1* deletion was constructed. A deletion cassette comprising the amdSYM marker cassette (Solis-Escalante et al., 2013) flanked by 60 bp homologous sequence to the *SeFDC1* promoter and the *SePAD1* terminator was amplified and transformed in *S. eubayanus* CBS 12357 (**Figure 1A**). Typically targeted deletion occurs through double cross-over mediated by homologous recombination and directed by the flanking homologous sequences. After transformation, acetamide growing transformants were genotyped by PCR for both the presence of the deletion cassette at the *SePAD1- SeFDC1* locus and for the presence of the wild type locus. All transformants were heterozygote harboring both versions of the locus [deleted (485 bp) and wild type (1027 bp)] (**Figure 1B**). One transformant was renamed IMK747 (**Figure 1B**). IMK747 was sporulated and tetrads were micro-manipulated. All four spores were growing on SMG medium but replica-plating on acetamide led to a two:two segregation of the amdSYM marker (**Figure 1C**) confirming the heterozygote *PAD1-FDC1* locus in IMK747 (*MATa/MATα*Se*pad1-Sefdc1*Δ*::*amdSYM*/SePAD1-SeFDC1*). As most wild type strains, *S. eubayanus* CBS 12357 is homothallic, in other words after spore germination haploids have the capability to switch mating type and mate with itself to form a stable diploid stage. Therefore spore S1 (**Figures 1B,C**) was grown on YPD plates and subsequently streaked out to separate single colony isolates. They were sequentially grown on SMG plates and replica plated on SM-Ace medium. Acetamide growing isolates were confirmed by PCR to only carry the deleted version of the *SePAD1- SeFDC1* locus. Subsequently, the PCR confirmed isolates were shown to be diploid by flow cytometry. A strain fulfilling all these characteristics (homozygote deleted *SePAD1-SeFDC1* locus; diploid genome) was selected and renamed IMK749 (*MATa/MATα Sepad1-Sefdc1Δ::*amdSYM*/Sepad1-Sefdc1Δ::*amdSYM).

**FIGURE 2:**
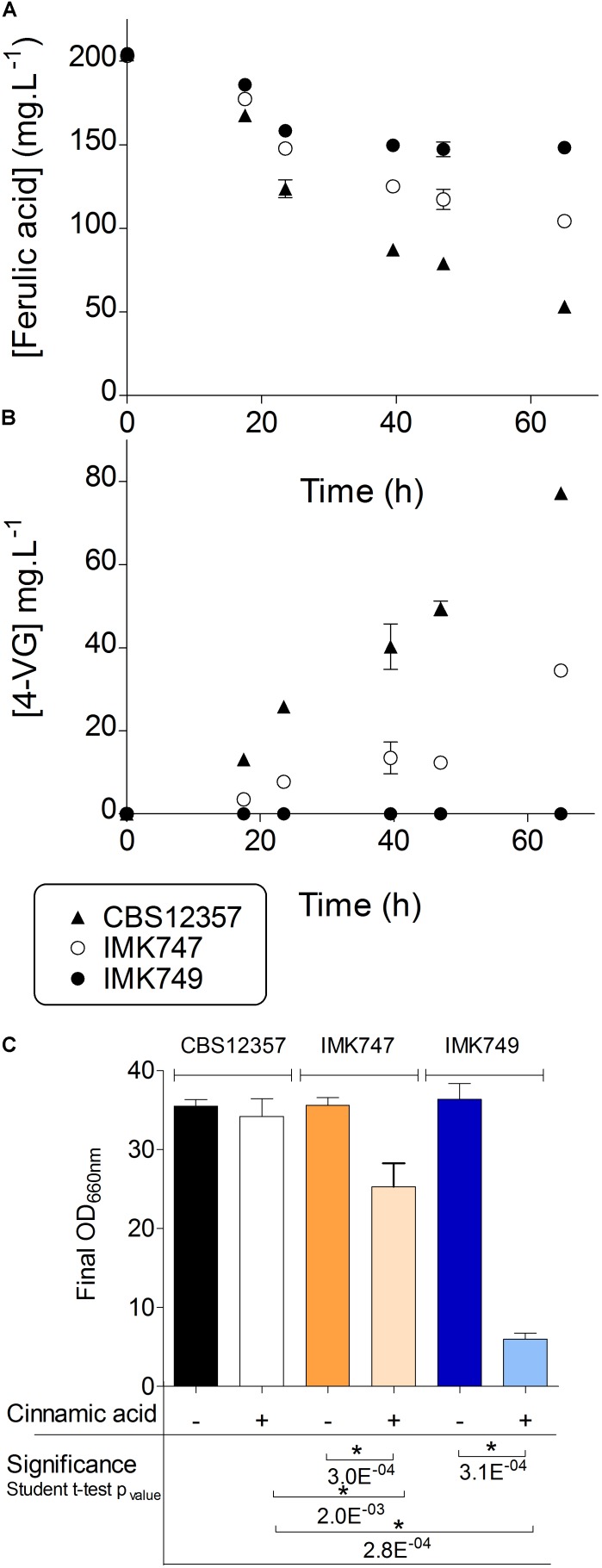
Ferulic acid conversion and cinnamic acid sensitivity of *PAD1-FDC1* deletion strains. *S. eubayanus* CBS 12357 (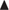, *SePAD1-SeFDC1* / *SePAD1-SeFDC1*, Pof^+^), IMK747 (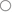, *Sepad1-Sefdc1*Δ::amdSYM / *SePAD1-SeFDC1*, Pof^+^) and IMK749 (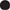, *Sepad1-Sefdc1*Δ::amdSYM / *Sepad1-Sefdc1*Δ::amdSYM, Pof^-^) were grown in SWM-fa in 24-deepwell plates at 20°C. The cultures were sampled regularly and supernatants were analyzed for ferulic **(A)** and 4-VG **(B)** concentrations. The same three strains were grown in SWM and SWM-ca in 24-deepwell plates at 20°C. Final optical densities at 660 nm were compared **(C)**. Variation in final optical density between the two conditions was evaluated by Student’s *t*-test statistics (significance threshold *p* = 0.01). The data presented show average ± standard deviation of independent triplicate experiments. ^∗^ denotes statistically significant differences.

The ability to convert ferulic acid into 4-VG was tested in the single heterozygote *SePAD1-SeFDC1* knockout IMK747 and the double *FDC1-PAD1* knockout IMK749 and compared to the parental strain *S. eubayanus* CBS 12357 (**Figure 2**). Cultures were grown in 24-deepwell plates in synthetic wort medium containing 1 mM ferulic acid (SWM-fa). The CBS 12357 strain consumed about 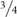 of the supplied ferulic acid (**Figure 2A**). The single knockout strain IMK747 showed a ferulic acid consumption that was approximately half of CBS 12357, while in the double *PAD1-FDC1* knockout IMK749 the ferulic acid concentration dropped by only 13% (**Figure 2A**). In strains CBS 12357 and IMK747 the consumed ferulic acid was nearly all stoichiometrically converted into 4-VG (**Figure 2B**). The strain IMK749 was not exhibiting any sign of 4-VG conversion and this in spite of a slight drop in extracellular concentration of ferulic acid.

### *S. eubayanus* IMK749 Exhibited an Exacerbated Sensitivity to Cinnamic Acid

Naturally occurring hydroxycinnamates like ferulic acid and cinnamic acid have been shown to inhibit growth of *S. cerevisiae* (Baranowski et al., 1980; Clausen et al., 1994; Larsson et al., 2001). Indeed the protonated form of weak organic acids (*e.g.*, ferulic and cinnamic acid) can freely diffuse across the membrane. Entering the cell the acid dissociates liberating a proton and therefore affecting intracellular pH. The protons pumped out to counter the lowering pH are expelled at the expense of ATP creating an energy expenditure futile cycle (Mollapour et al., 2004; Hazelwood et al., 2006; Piper, 2011). In contrast, the import of 4-VG or styrene (decarboxylated product of cinnamic acid) should not result in accelerated acidification of cytosol and would not require extra ATP investment. Therefore cells having lost the ability to decarboxylate hydroxycinamates should exhibit a stronger growth inhibition. To test this characteristic, the *S. eubayanus* CBS 12357, IMK747 (*Sepad1-Sefdc1Δ*::amdSYM/*SePAD1-SeFDC1*) and IMK749 (*Sepad1-Sefdc1Δ*::amdSYM/*Sepad1-Sefdc1Δ*::amdSYM) were grown in synthetic wort medium containing 1 mM cinnamic acid (SWM-ca). On SWM all strains showed comparable growth. Conversely, in SWM-ca, growth of the double *PAD1-FDC1* knockout IMK749 was almost completely abolished, while the single knockout showed a significant reduced growth compared to the parental strain CBS 12357 (Student *t*-test *p*-value < 0.01) (**Figure 2C**) confirming the role of *PAD1-FDC1* in the conversion and detoxification of growth inhibiting cinnamic acid into styrene.

To adapt the assay to high-throughput testing, the effect of the cinnamic acid was evaluated based on biomass yield. The Cinnamic Acid Sensitivity Index (CASI) was calculated as the ratio of the optical density (OD_660nm_) of the culture grown in presence of 1 mM of cinnamic acid over OD of the culture without cinnamic acid at the end of growth. The parental strain showed a low CASI of 0.1, while the heterozygote single *Sepad1-Sefdc1Δ* knock-out strain IMK747 showed a CASI of 23, and the homozygote double *Sepad1-Sefdc1Δ* knock-out IMK749 a CASI of 73, showing a high cinnamic acid sensitivity (**Figure 2C**).

### A Fast and Reliable UV-Vis Method for Ferulic Acid and 4-Vinyl-Guaiacol Determination

**FIGURE 3:**
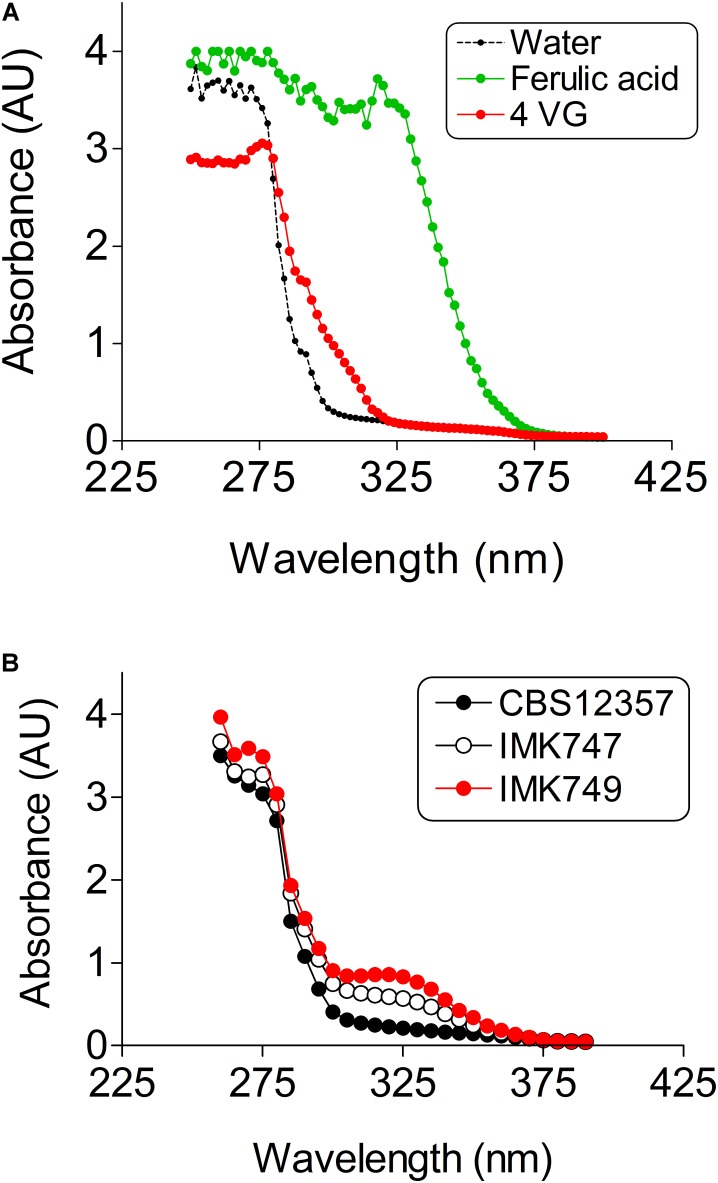
UV-Vis absorptivity of ferulic acid, 4-vinylguaiacol (4-VG) and air. **(A)** Absorption spectra of wavelengths ranging from 250–400 nm of 1 mM ferulic acid (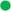), 1 mM 4-VG (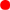) and water (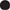) were determined in 96-well microtiter plates in a Tecan Infinite M200Pro spectrophotometer. The data presented show average ± standard deviation of eight independent replicate experiments. **(B)** Absorption spectra of wavelengths ranging from 250 nm to 400 nm of *S. eubayanus* CBS 12357 (*SePAD1-SeFDC1*/*SePAD1-SeFDC1*, Pof ^+^) (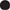), IMK747 (*Sepad1-Sefdc1*Δ::amdSYM / *SePAD1-SeFDC1*, Pof ^+^) (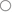)and IMK749 (*Sepad1-Sefdc1*Δ::amdSYM/*Sepad1-Sefdc1*Δ::amdSYM, Pof^-^) (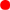). Cells were grown in SWM-fa at 20°C for 72 h. Absorption spectra were determined of five times diluted supernatants in a Tecan Infinite M200Pro spectrophotometer.

Ferulic acid, cinnamic acid and 4-VG can be measured by HPLC methods that entail up to 30 min analysis per sample (*e.g.*, Bourne et al., 2000; Koopman et al., 2012; Vos et al., 2015) a timing that is not compatible with the analysis of several thousand of samples. Therefore a new high-throughput compatible method was required. The structure difference between ferulic acid and 4-VG is restricted to a single carboxylic group. Although saturated carboxylic groups absorb very weakly in UV-Vis, their absorptivity significantly increases when the carboxylic group is conjugated to an alkenyl function (C = C) like in the configuration of ferulic acid. UV-Vis absorption spectra between 200 and 400 nm of ferulic acid and 4-VG revealed a clear difference at wavelengths higher than 280 nm. The 4-VG spectrum exhibited a sharp decrease to reach 95% reduction at 300 nm while ferulic acid absorption remained maximal till 335 nm (**Figure 3A**). The difference in absorption characteristics enabled a high-throughput analysis of culture media in 96-well plates in a microplate spectrophotometer, which reduced sample analysis time to a couple of seconds.

The conversion capacity of ferulic acid into 4-VG in the single *PAD1-FDC1* knockout IMK747 and the double *PAD1-FDC1* knockout IMK749 was compared to the parental strain CBS 12357. The *S. eubayanus* strains CBS 12357 (Pof^+^), IMK747 (Pof^+^) and IMK749 (Pof***^-^***) were grown in SWM-fa (**Figure 3B**). The absorption spectra of supernatant of 3 days old cultures were determined. The parental strain CBS 12357 spectrum showed a clear decrease in the peak at 330 nm compatible with the conversion of ferulic acid into 4-VG. The single knockout strain showed a reduced diminution of the ferulic acid peak, while no decrease of the peak height at 330 nm was noticeable in the double knockout and the spectrum remained undistinguishable to that of the SWM control spectrum (**Figure 3**).

### Screening for UV-Mutagenized Strains With Reduced 4-VG Formation

Although the deletion of the *PAD1-FDC1* gene pair was sufficient to explain the genetic basis of a Pof***^-^*** phenotype, the reluctance to apply GM strains like IMK749 (*Sepad1-Sefdc1Δ::*amdSYM/*Sepad1-Sefdc1Δ::*amdSYM) in construction of hybrids that could potentially be used in production of commercial beverages restricted the range of mutagenesis methods to approaches often referred to as “classical strain improvement” and more specifically introduction of mutations through UV irradiation and screening of a wanted phenotype. Random mutagenesis by UV-light has been reported to cause a wide range of changes in the genome sequence of yeast cells, including transitions and transversions (Abdulovic and Jinks-Robertson, 2006), generating strains with a wide variety of properties.

A method to select the strains with the required properties after random mutagenesis was developed. First, general properties of the strains were assessed which comprised the determination of growth and fermentation capacity. Random mutagenesis may be a drastic method that can easily have a negative impact on the general characteristics of the yeast strain. Second, high throughput methods were applied to screen and select yeast strains which have a reduced ability (or lack the ability) to convert ferulic acid into 4-VG, as determined by the cinnamic acid sensitivity index (CASI) and the ferulic acid conversion index (FACI).

**FIGURE 4:**
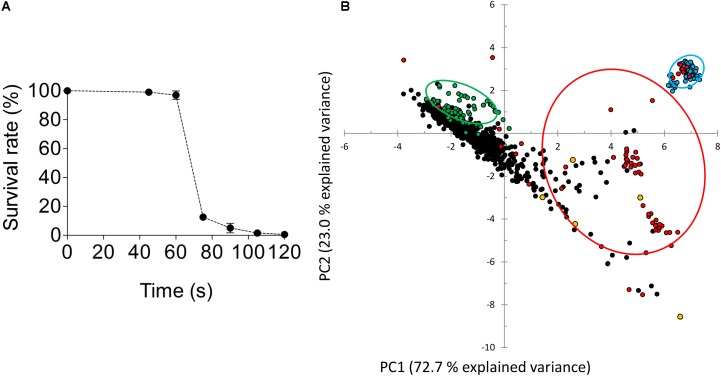
Screening of a mutagenized population of *S. eubayanus* CBS 12357. **(A)** Survival after UV exposure of CBS 12357 spores. Plated spores were mutagenized using a UVC-lamp, 36W, MSC-Advantage Biological Safety Cabinet, Thermo Fisher Scientific. Plates were subjected to UV for different periods ranging from 45–120 s. Surviving germinated spores were quantified after 5 days of incubation at 20°C in the dark. Spores sample exposed to UV for 80 s were further processed. **(B)** Principal component analysis (PCA) two-dimensional score plot of parental strains CBS 12357, UV mutagenized strains, and culture medium from cultivations in 96-well microtiter plates. About 1000 picked colonies were arrayed in 96 MTP format. The mutagenized population was further inoculated in SWM, SWM-ca and SWM-fa. Principal Component Analysis (PCA) was performed on a dataset comprising CASI readouts as determined by final OD_660nm_ values after 72 h growth on SWM and SWM-ca, and FACI readouts as determined by discrete UV-Vis absorbance values from 280–360 nm with 10 nm intervals of SMW-fa culture supernatants after 72 h of cultivation. Ellipses were drawn to include 68% of the particular subgroup of cultures. The 80 replicates of the parental strain *S. eubayanus* CBS 12357 were shown in green, the blank culture replicates (no cells) were shown in blue, the UV-mutagenized variants were shown in black and the UV mutagenized strains exhibiting a fitness reduction were shown in red.

To enhance the chance of success, spores, which only contained a single copy of each chromosome, of the *S. eubayanus* strain CBS 12357 were UV-mutagenized for 80 s, an exposure that resulted in a 1% survival rate that was shown to yield improved (recessive) phenotypes in industrial yeast (Hashimoto et al., 2005) (**Figure 4A**). Emerging colonies were arrayed in 96-well MTP format and grown on SWM. This culture served to inoculate three different media. SWM and SWM-ca (1 mM cinnamic acid) cultures were used to assess the CASI. The third culture (SWM-fa) was used to measure FACI by measuring the UV-Vis absorption spectrum of the culture supernatants. A total of ca. 1000 mutagenized colonies were tested (**Figure 4B**) and data points including growth on SWM and SMW-ca approximated by OD_660nm_ after 72 h of cultivation, discrete UV-Vis absorbance ranged from at 280–380 nm with 10 nm intervals of SMW-fa culture supernatant after 72 h of cultivation were statistically evaluated by PCA (**Figure 4B**). Three groups were pre-labeled in this analysis, (i) the first one included replicates (*n* = 80) of the parental strain CBS 12357 (in green **Figure 4B**), (ii) the second include blank cultures (*n* = 80) (in blue **Figure 4B**) and (iii) the third group included variants exhibiting a pronounced growth defect on SMW defined by an OD_660nm_ after 72h 25% lower that the average growth (OD_660nm_ at 72 h) of CBS 12357 (in red **Figure 4B**). By selecting the first two components that together explained more than 95% of the data variance, the variants formed a group distinct from the parental CBS 12357 strain group revealing the difference in preculture conditions between the parental strain and the UV mutagenized variants. The poor growing cell lines were spread over three quadrants. Although not clearly forming a centered group, about fifty UV strains were separated in the bottom right quadrant. Five different UV mutagenized variants from that quadrant were selected two exhibited CASI and FACI scores comparable to *S. eubayanus* IMK749 (CASI^IMK749^ = 72.7 and FACI^IMK749^ = 15.7) while the other three showed deviation from IMK749 values (indicated in yellow **Figure 4B** and **Table 3**). As comparison, wild type CBS 12357 which converts ferulic acid into 4-VG, had a CASI value of 0.1 and a FACI of 73. The five mutagenized variants exhibited CASI higher than 5 and FACI value ranged between 60 (for HTSE022) and 15 (for HTSE037 and HTSE042).

**Table 3:** CASI and FACI performance indicators of the parental *S. eubayanus* strain CBS 12357, of single and double *PAD1-FDC1* deletion mutants IMK747 and 749, and of five UV-mutagenized isolates.

*S. eubayanus* strain	Relevant characteristics	CASI	FACI
CBS 12357	*MATa/MATα SePAD1-SeFDC1/SePAD1-SeFDC1*) Pof^+^	0.1	72.7
IMK747	*MATa/MATα Sepad1-Sefdc1Δ::amdS/SePAD1-SeFDC1* Pof^+^	22.7	36.7
IMK749	*MATa/MATα Sepad1-Sefdc1Δ::amdS/Sepad1-Sefdc1Δ::amdS* Pof^-^	72.7	15.7
HTSE022	*MATa/MATα SePAD1-SeFDC1/SePAD1-SeFDC1* (UV mutagenised) Pof^+^	11.8	59.9
HTSE023	*MATa/MATα SePAD1-SeFDC1/SePAD1-SeFDC1* (UV mutagenised) Pof^+^	5.7	51.9
HTSE033	*MATa/MATα SePAD1-SeFDC1/SePAD1-SeFDC1* (UV mutagenised) Pof^+^	12.7	42.9
HTSE037	*MATa/MATα SePAD1-SeFDC1/SePAD1-SeFDC1* (UV mutagenised) Pof^-^	60.6	15.7
HTSE042	*MATa/MATα Sepad1-Sefdc1Δ/ Sepad1-Sefdc1Δ*^∗^ (UV mutagenised) Pof^-^	68.3	15.4


*The UV mutagenized isolates (HTSE022-023, HTSE033, 37 and 42) were grown in 96-well microtiter plates filled with SWM at 20°C for 48 h in an orbital incubator (Infors Multitron) at 250 rpm. Subsequently, the microtiter plates were replica-plated in three different media by transferring 10 μl of each culture into fresh microtiter plates filled with either 200 μL SWM or SWM-fa or SWM-ca. The reference strain S. eubayanus CBS 12357, IMK747 and 749 were grown in shake flask filled with either 100 mL SWM or SWM-fa or SWM-ca. CASI and FACI indicators were calculated as described in the Materials and Methods section. ^∗^Indicates *pad1-fdc1* deletion resulting from UV mutagenesis.*

### Characterization of *S. eubayanus* Strains With a Reduced Ability to Produce 4-VG

**FIGURE 5:**
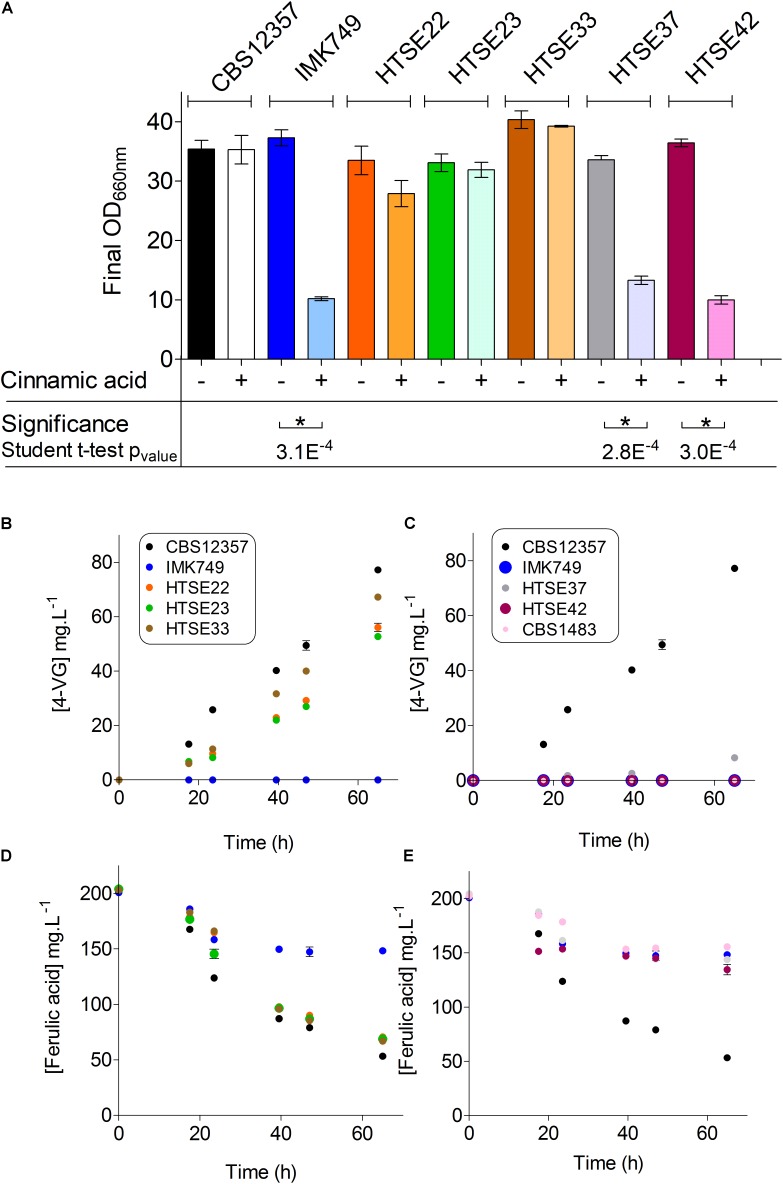
Characterization of UV variants expressing improved phenotype. **(A)** Cinnamic acid sensitivity assay. Five selected variants (HTSE022, HTSE023, HTSE033, HTSE037, and HTSE042) and control strains CBS1257, IMK747, and IMK749 were grown in 24-deepwell plates in 3 ml SMW and SMW-ca. Growth and final OD_660nm_ was recorded and differential growth was statistically evaluated by a Student’s *t*-test. Significance threshold was set at 0.01. The data presented show average ± standard deviation of three independent replicate experiments. **(B)** 4-VG concentration profiles of CBS 12357 (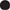), IMK749 (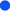), HTSE022 (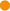), HTSE023 (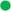), HTSE033 (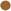) cultivated in 24-deepwell plates in 3 mL SWM-fa. **(C)** 4-VG concentration profiles of CBS 12357 (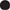), IMK749 (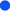), HTSE037 (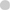), HTSE042 (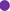) and *S. pastorianus* CBS1483 (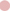) grown in 24-deepwell plates in 3 mL SWM-fa. **(D)** Consumed ferulic acid in SWM-fa by CBS 12357, IMK749, HTSE022, HTSE23, and HTSE33. **(E)** Consumed ferulic acid in SWM-fa by CBS 12357, IMK749, HTSE037, HTSE042 and *S. pastorianus* CBS 1483. The colored code used in panels D and E is identical to that used in panels **(D)** and **(E)**. The data presented show average ± standard deviation of independent triplicate experiments.

The set of five selected *S. eubayanus* CBS 12357 variants was compared to the parental *S. eubayanus* CBS12537 and the control *PAD1-FDC1* deletion strains *S. eubayanus* IMK747 (*MATa/MATαSepad1-Sefdc1Δ::*amdSYM*/SePAD1-SeFDC1*) and *S. eubayanus* IMK749 (*MATa/MATα Sepad1-Sefdc1Δ::*amdSYM*/Sepad1-Sefdc1Δ::*amdSYM) in shake flask cultures. Three of the selected variants HTSE022, HTSE023 and HTSE033 did not show significant cinnamic acid inhibition relative to the parental strain CBS 12357 (*MATa/MATα SePAD1-SeFDC1/ SePAD1-SeFDC1*) and IMK747 (*MATa/MATα Sepad1-Sefdc1Δ::*amdSYM*/SePAD1-SeFDC1*). This confirmed that the values measured in the screening could be benchmarked to the those of the controls CBS 12357 and IMK747. Conversely, variants HTSE037 and HTSE042 showed strong and significant growth inhibition (Student’s *t*-test *p*-value = 2.8E^-4^ and 3.0E^-4^ respectively) compared to the parental strain CBS 12357. These data were highly comparable to those of IMK749 (*MATa/MATα Sepad1-Sefdc1Δ::*amdSYM*/ Sepad1-Sefdc1Δ::*amdSYM) (**Figure 5A**).

In line with the toxicity assay, the selected variants HTSE022, HTSE023 and HTSE033 showed formation of 4-VG comparable to that of the parental strain *S. eubayanus* CBS 12357 (**Figure 5B**). Further corroborating the toxicity assay results, the variants HTSE037 and HTSE042 did not convert ferulic acid into 4-VG, a result that perfectly matched the performance of the double *PAD1-FDC1* knockout IMK749 (**Figure 5B**).

### Genome Sequence Analysis

**FIGURE 6:**
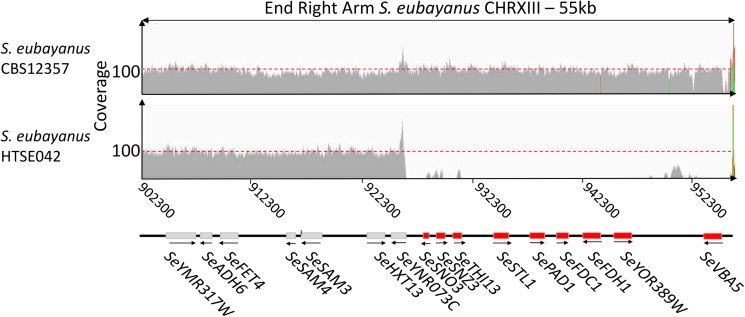
Sequence comparison of parental strain *S. eubayanus* CBS 12357 with UV-mutagenized variant HTSE042. Sequencing read mapping of *S. eubayanus* reference CBS 12357 and UV-variant HTSE042 strain onto CBS 12357 assembly (PRJNA243390) (Baker et al., 2015). The figure represents local mapping at the end of right arm of *S. eubayanus* CHRXIII. Both strains showed a similar coverage of the region (ca 95-100-fold). HTSE042 showed an absence of read mapping at the chromosomal region between *S. eubayanus* ortholog of *S. cerevisiae* YNR073C renamed *Se*YNR073C and *SeVBA5* located at the end of *Se*CHRXIII. Reads were mapped using Burrows-Wheeler Aligner BWA (Li and Durbin, 2010). The resulting bam files were visualized using IGV (http://software.broadinstitute.org/software/igv/).

Out of the two Pof^-^ selected variants, HTSE042 exhibited the best performance. While HTSE037 showed an significant reduction of produced 4-VG (Student’s *t*-test-value = 5.8E^-6^, **Figures 5A,B**), a low amount could still be detected, this was contrasting with the complete absence of 4-VG detection in HTSE042, therefore *S. eubayanus* variant HTSE042 was whole genome sequenced and compared to the parental strain *S. eubayanus* CBS 12357 genome assembly. Single-nucleotide variations and indels were determined using Pilon (Walker et al., 2014) and visualized using IGV^6^. Only 39 single nucleotide variations (SNV) were detected in HTSE042 sequence relative to CBS 12357. Theses SNV were distributed in coding regions for three of them and the remaining 36 were located in intergenic regions (at least 1 kb upstream and downstream of a coding sequence). Out of the three mutations within coding sequences only one resulted in an amino acid change (*SePST1*, G-225-S), the other two mutations in *RSP5* and *SUN4* were synonymous mutations. However, the most severe and relevant mutation was a large deletion observed toward the right telomere of chromosome XIII. A region of approximately 27 kb was deleted in HTSE042 (**Figure 6**). This region harbored the genes *SePAD1* and *SeFDC1*. This is in contrast to the location of *PAD1-FDC1* in *S. cerevisiae* where the two genes are positioned close to the subtelomeric region of chromosome IV right arm.

### Characterization of Hybrids With a Reduced Capacity to Produce 4-VG

**FIGURE 7:**
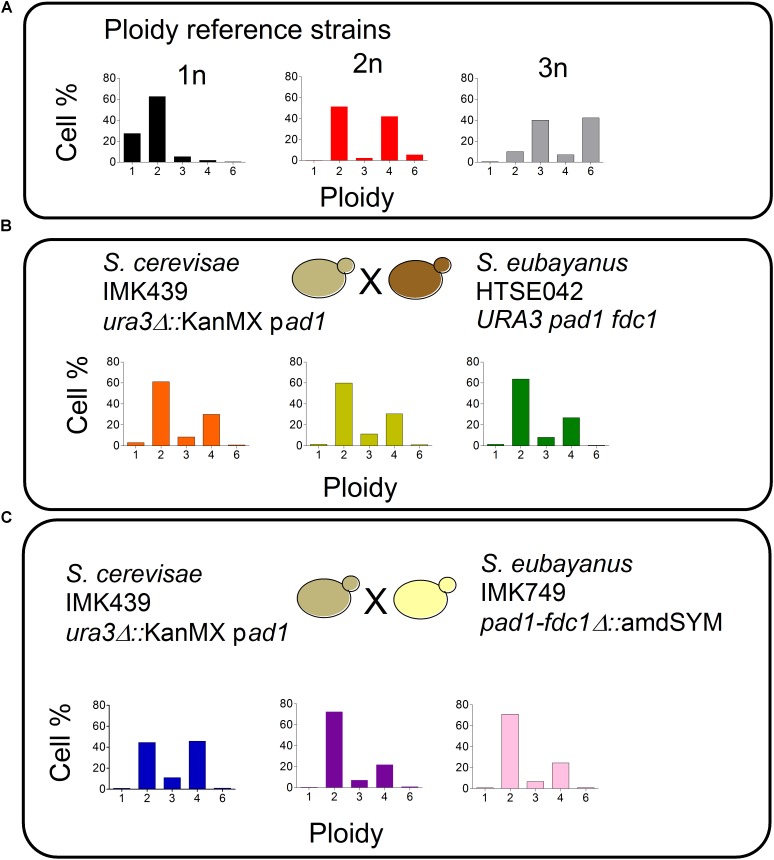
Ploidy estimation of *S. cerevisiae* × *S. eubayanus* hybrids. **(A)** Ploidy of control strains 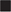 CEN.PK113-7D (1n), 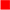 CEN.PK122 (2n), 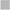 FRY153 (3n). **(B)** Ploidy estimation of *S. cerevisiae* (IMK439) × *S. eubayanus* (HTSE042) hybrids 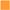 HTSH009, 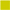 HTSH010 and 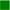 HTSH011. **(C)** Ploidy estimation of *S. cerevisiae* (IMK439) × *S. eubayanus* (IMK749) hybrids 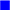 HTSH012, 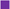 HTSH013 and 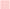 HTSH014. Cells were stained with SYTOX Green Nucleic Acid and analyzed on a flow cytometer equipped with a 488 nm laser.

*Saccharomyces pastorianus* strains are hybrids of *S. cerevisiae* and *S. eubayanus* parents. Therefore to form *de novo* hybrids, the screened Pof***^-^*** *S. eubayanus* variant HTSE042 was crossed with the *S. cerevisiae* strain IMK049. This strain derived from the CEN.PK lineage carried a non-functional *PAD1* allele (Otero et al., 2010; Nijkamp et al., 2012; Salazar et al., 2017). In addition to the absence of 4-VG negative (Pof***^-^***) phenotype and to ease the selection of the constructed hybrids, *S. cerevisiae* IMK439 was also carrying a *ura3Δ* allele interrupted by a KanMX cassette conferring resistance to G418 (*ura3Δ*::KanMX). Mass mating (Hawthorne and Philippsen, 1994) between spores of *S. eubayanus* HTSE042 and cells of *S. cerevisiae* IMK439 was performed for 5 h at 30°C. The mating mixture was then plated on minimal medium containing G418 (SMUG). The selection of true hybrids was based on their uracil prototrophy and G418 resistance, contributed by the *S. eubayanus* and *S. cerevisiae* parents, respectively. Based on specific typing PCR able to discriminate *S. cerevisiae* and *S. eubayanus* species, and ploidy determination by flow cytometry (**Figure 7**), three hybrid isolates were selected and named HTSH009, HTSH010, and HTSH011. In order to benchmark the performance of HTSH009 to 011, hybrids between *S. cerevisiae* IMK439 and *S. eubayanus* IMK749 that carried a deletion of *FDC1-PAD1* were also constructed and similarly three characterized hybrids renamed HTSH012 to 014 were selected. These six newly constructed hybrids were additionally compared to IMS0408 a diploid hybrid made from *S. eubayanus* CBS 12357 the parent of HTSE042 and IMK749 and *S. cerevisiae* IMK439 (Hebly et al., 2015).

**FIGURE 8:**
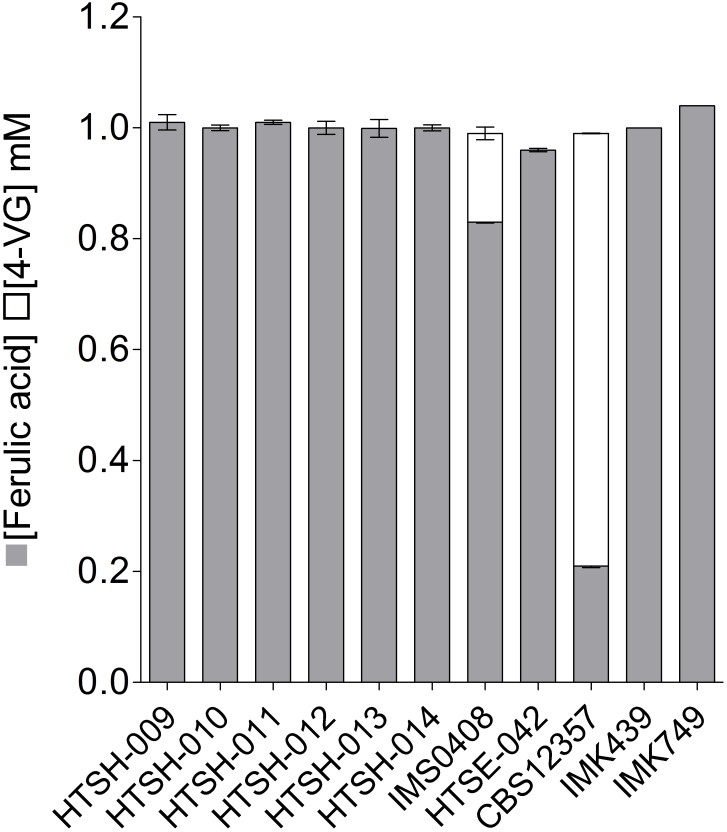
Ferulic acid consumption and 4-VG production of *S. cerevisiae* × *S. eubayanus* hybrids and parental strains. *S. cerevisiae* strain IMK439, *S. eubayanus* CBS 12357, IMK749 and HTSE042 and interspecies hybrids IMS0408, HTSH009-014 were grown micro-aerobically in 100 ml septa flasks with gas outlet containing 60 ml SWM-fa at 12°C. After 110 h ferulic acid (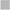) and 4-VG (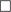) were determined by HPLC. The data presented show average ± standard deviation of independent triplicate experiments.

**FIGURE 9:**
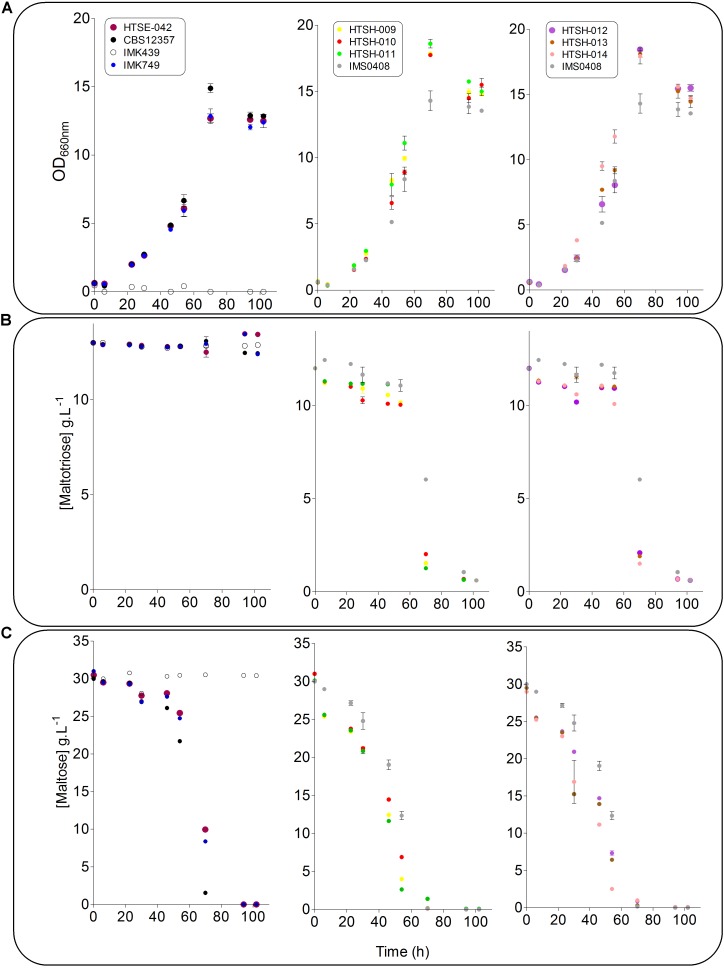
Fermentation performance of *S. cerevisiae* × *S. eubayanus* hybrids and parental control strains. *S. cerevisiae* strain IMK439 (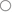), *S. eubayanus* CBS 12357 (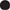), IMK749 (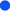) and HTSE042 (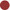) and interspecies hybrids IMS0408 (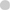), HTSH009 (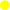), HTSH010 (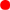), HTSH011 (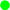), HTSH012 (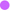), HTSH013 (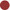) and HTSH014 (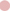) were grown micro-aerobically in 100 ml septa flasks with gas outlet containing 60 ml SWM-fa at 12°C. OD_660nm_ **(A)**, maltotriose **(B)** and maltose **(C)** were measured regularly for 110 h. The data presented show average ± standard deviation of independent triplicate experiments.

To validate the strategy, the hybrids labeled HTSH009 to 014 were grown in SWM-fa under micro aerobic conditions at 12°C and sugar consumption, ethanol production ferulic acid and 4-VG production were measured (**Figure 8**). As previously shown, the *S. eubayanus* CBS 12357 strain did not consume maltotriose (Hebly et al., 2015; Krogerus et al., 2015). This phenotype was logically shared with the IMK749 and HTSE042 strains. The parental *S. cerevisiae* strain IMK439 auxotrophic for uracil expectedly did not grow on SWM-fa medium. Interestingly, all newly constructed hybrids grew faster than their parents and the hybrid IMS0408. They also grew to higher OD_660nm_, this was related to the capacity of the hybrids (HTSH009-014 and IMS0408) to consume all sugars including maltotriose present in the culture resulting in a significant increase (+25%, Student *t*-test *p*-value = 2.19E^-05^) in ethanol concentration at the end of the fermentation. But no significant differences were observed between the three groups of constructed hybrids regarding growth kinetics and ethanol production but conversely, the difference in 4-VG was significant between the hybrid groups. The hybrid between wildtype *S. eubayanus* CBS 12357 and *S. cerevisiae* IMK439, IMS0408 (Hebly et al., 2015) consumed ferulic acid and converted it to 4-VG stoichiometrically (**Figure 8**). The hybrids issued from the mating of either *S. eubayanus* IMK749 or HTSE042 were not distinguishable and exhibited a complete absence of 4-VG synthesis (**Figures 8**, **9**). These results demonstrated the applicability of the UV-mutagenized Pof^-^ variant to construct *de novo* hybrids holding phenotypes potentially relevant for the brewing industry.

## Discussion

The results reported in this study described a methodology to successfully construct Pof***^-^*** strains which turned out to be valuable to generate *de novo S. pastorianus* interspecies hybrids (Nakao et al., 2009; van den Broek et al., 2015). While 4-vinylguaiacol (4-VG) and 4-vinylphenol (4-VP) are of key importance in top-fermented wheat beers, this distinctive clove-like and phenolic aroma is highly undesired in lager beers. Compared to other classes of flavor molecules found in beverages, 4-VG exhibits a lower sensory threshold value, reported to be 0.3 mg⋅L^-1^ (Vanbeneden et al., 2007) and the typical concentration conveying a pleasant aroma in wheat beer ranges from 2 to 3.5 mg⋅L^-1^. Above 4 mg⋅L^-1^, 4-VG negatively affects the bouquet of top-fermented wheat beers. Consequently, low concentrations of 4-VG might modify or hide the perception of other flavors and might compromise development of beverage products with innovative and more diverse aroma profiles (Gallone et al., 2016). Therefore availability of yeast strains not only reduced but devoid of their ability to produce 4-VG would be seen as valuable asset in mating programs. Although, the biochemistry of the 4-VG formation is well understood, implementation of mutation leading to deletion/interruption of either *PAD1* or *FDC1* or both genes by targeted genetic engineering [*e.g.*, CRISPR genome editing (de Vries et al., 2017)] cannot be envisaged in the context of beverage production (Varzakas et al., 2007; Twardowski and Malyska, 2015). Traditionally food and beverage industries relied on induction of random mutations by chemical or physical mutagens (Giudici et al., 2005), evolutionary engineering (Ekberg et al., 2013; Brickwedde et al., 2017) and mating approaches (Steensels et al., 2014; Hebly et al., 2015; Magalhaes et al., 2017) and subsequent selection of phenotypically improved lineages. As often quoted the first law of screening ‘you get what you screen for’ associates the importance of the assays accuracy relative to the screening targets. To warrant the selection of true positive candidates, two complementary assays were used in this study. The cinnamic acid sensitivity assay served two purposes, (i) to exclude strains with fitness reduction and (ii) select for variants responding negatively to weak acid stress, a trait that hypothetically could reflect an impaired detoxification via decarboxylation. The second test assessed qualitatively the ferulic acid conversion in 4-VG. The quantification of proxy value as CASI and FACI values (**Figure 3**) enabled to rank and select mutagenized colonies that had conserved sufficient growth with reduced 4-VG biosynthesis and therefore from a very early stage limit testing false positives. The value and efficiency of this bimodal screening approach was illustrated by the high selection yield of true positive variants. Typically a mutagenesis and screening program would encompass testing of a large population of cells that should in theory be proportional to the genome gene density and size. It is not exaggerated to have to screen populations of size ranging from 6,000 to 20,000 mutagenized yeast isolates to find a hand full of potential variants. The 4-VG negative screening proved to be extremely sensitive as two Pof^-^ variants were accurately sorted in the first screening level within a crowd of only ca. 2000 mutagenized colonies. With such efficiency transferring this phenotypic trait to other *S. eubayanus* strains and further to other yeast that might be used in mating approaches would be realistic. Hitherto most hybridization involving *S. eubayanus* rested on the use of the strain CBS 12357 originating from Patagonia (Libkind et al., 2011). Since its first isolation by Libkind et al. (2011) *S. eubayanus* has been isolated on several continents (Bing et al., 2014; Peris et al., 2014; Gayevskiy and Goddard, 2016) but so far the information about these isolates remains limited, however inspection of available genome sequences confirmed the presence of the two genes *PAD1* and *FDC1* suggesting that these isolates would be able to produce 4-VG. This approach could even be applied to *Saccharomyces cerevisiae* strains and more specifically on wild type isolates which have not yet lost the Pof^+^ trait under domestication evolutionary pressure (Gallone et al., 2016). Eventually, the approach could be applied on different *Saccharomyces* species known to harbor *PAD1* and *FDC1* orthologs as *S. paradoxus, S. kudriavzevii*, and *S. mikatae*.

The Pof^-^ *S. cerevisiae* × *S. eubayanus* hybrids described in this study were not the first to be reported. But contrasting with the rational design developed here, the precedent examples described Pof***^-^*** phenotypes that related to heterosis or hybrid vigor since Pof***^-^*** offspring were resulting from crosses involving Pof^+^ parents. These specific hybrids expressed a dominant Pof^-^ phenotype not yet completely understood in the context of these works. While it was not possible to exclude that the phenotype might still be linked to randomly occurring non-sense mutations in the *PAD1* and *FDC1* genes, it demonstrated that selection of Pof***^-^*** hybrids remained to a large extent accidental. The results reported in this work further emphasized the importance of a systematic methodology to easily and meticulously generate parental Pof^-^ parental strains that would significantly contribute to intensification of *de novo* lager yeast hybridization program.

## Author Contributions

JD, SW, JP, and J-MD designed the experiments. JD, SW, MB, and J-MD critically analyzed the results. JD and SW performed the experiments. JD and J-MD wrote the manuscript. All authors read and approved the final manuscript.

## Conflict of Interest Statement

JD, SW, JP, and J-MD have filed a patent application based on the findings of this study. The remaining author declares that the research was conducted in the absence of any commercial or financial relationships that could be construed as a potential conflict of interest.

## Footnotes

1 https://www.rstudio.com/

2 https://www.r-project.org/

3 http://github.com/vqv/ggbiplot

4 http://software.broadinstitute.org/software/igv/

5 https://www.ncbi.nlm.nih.gov/

6 http://software.broadinstitute.org/software/igv/
